# Improving the Usefulness and Use of Meta-Analysis to Inform Policy and Practice

**DOI:** 10.1177/0193841X241229885

**Published:** 2024-02-03

**Authors:** Rebecca Maynard

**Affiliations:** 1 Graduate School of Education, 6572 University of Pennsylvania, Philadelphia, PA, USA

**Keywords:** meta-analysis, generalizability, applicability, public policy, methodology, impact evaluation, program evaluation, policy evaluation, research synthesis

## Abstract

This chapter begins with an overview of recent developments that have encouraged and facilitated greater use of research syntheses, including Meta-Analysis, to guide public policy and practice in education, workforce development, and social services. It discusses the role of Meta-Analysis for improving knowledge of the effectiveness of programs, policies, and practices and the applicability and generalizability of that knowledge to conditions other than those represented by the study samples and settings. The chapter concludes with recommendations for improving the potential of Meta-Analysis to accelerate knowledge development through changing how we design, conduct, and report findings of individual studies to maximize their usefulness in Meta-Analysis as well as how we produce and report Meta-Analysis findings. The paper includes references to resources supporting the recommendations.

Sound public policy depends critically on access to and responsible use of credible evidence. Over the past 20 years, the U.S., Canada and many European countries have accelerated their production of evidence to support public policy design, implementation and monitoring and mandated greater use of evidence to inform decision-making (Commission on Evidence-Based Policy, 2017; Nutley et al., 2002; Van Woensel, 2021). Yet, there remain large gaps in the evidence base and shortfalls in its use due to issues of access to and quality of the evidence (Donaldson et al., 2015; Van de Goor et al., 2017). Research is time consuming and costly. Moreover, it typically requires multiple studies to provide credible evidence to support important, real-world policy decisions. This is because very few studies use sufficiently large and diverse samples and are implemented in sufficiently diverse contexts so support generalizing the available evidence on effectiveness to the particular context of interest.

Meta-Analysis is useful for pooling evidence from multiple studies to expand knowledge about the likely impacts of particular programs, policies or practices. When there are many studies, it also is an efficient way to explore the degree and predictors of impact variation across population groups and contexts—“generalizability” of findings. However, Meta-Analysis has limitations. Many studies produce evidence that falls short in terms of its credibility and/or accessibility to study details needed for inclusion in a Meta-Analysis. Moreover, differences in the programs or policies being evaluated, the populations targeted, and/or the measures used can complicate or preclude responsible generalizing of findings to contexts other that reflected by the weighted population groups, implementation conditions, and settings represented by the included study samples.

This chapter begins with an overview of recent developments that have encouraged and facilitated greater use of research syntheses, including Meta-Analysis. This includes growing interest in improving the ability to generalize from study findings to varied target populations and conditions of interest (Tipton, 2014; Tipton & Matlen, 2019; Tipton & Olsen, 2018, 2022). It then discusses ways to optimize the usefulness and use of Meta-Analysis to guide the design and implementation of social policies and programs. The chapter concludes with recommendations for improving the potential of Meta-Analysis to accelerate knowledge development through changing how we design, conduct and report findings from primary evaluations and, subsequently, synthesize these findings narratively and using Meta-Analysis. The aim is to create an eco-system where systematic reviews and Meta-Analyses can be routinely updates as new evidence emerges, thereby expanding our understanding of what we know and what we need to learn about the effectiveness of particular programs, policies, or practices. This includes what we know about the bounds on generalizability and applicability of existing evidence.

## The Push for More and Better Evidence to Guide Policy Development and Implementation

Recent shifts in the policy arena have stimulated advancements in the quantity and quality of evidence on the impacts of various programs, policies, and practices and strengthened commitments of and support for policy makers and practitioners to use evidence. Increasing numbers of funders and shares of their financial support are shaped by evidence on expected impacts, returns on investment (benefits/costs), and efficiency gains (cost-effectiveness). For example, it is now common for federal and state funded programs and policies to require evidence of their expected benefits (Baron, 2018; Pew Trusts & MacArthur Foundation, 2014; Stack, 2018). Three prominent examples are the U.S. Department of Education’s $50 million investment in its Post Secondary Success Grant program to “equitably improve …student outcomes by …implementing, scaling, and rigorously evaluating evidence-based activities…,”^1^ its $60 million investment in evidence-based efforts to strengthen the pipeline of K-12 teachers (U.S. Department of Education, 2022) and its $679 million in funding to 67 “Investing in Innovation” (I-3) grantees to designed, implement, and evaluate a broad range of education innovations that (Boulay et al., 2018)*.^2^ Yet even where evidence is required, the quality and coverage often fall short on the credibility scale. Within each cluster of studies, there was intentional variation in focal program priorities, program components, mediators, and focal outcomes.

In an ideal world, policymakers would have access to a large body of evidence from multiple studies conducted in varied contexts and synthesized in ways that inform decisions about the likely costs and benefits of recommended options. When there are few relevant studies, the syntheses may entail only a narrative description of the nature of the evidence—including strengths and weaknesses—supported by summary tables. When there are many studies of similar programs, policies or practices, the syntheses can include statistical analyses that pool findings across studies using Meta-Analysis (Alexander, 2020; Borenstein, 2019; Borenstein et al., 2015; Lipsey & Wilson, 2001).

**Figure 1.**
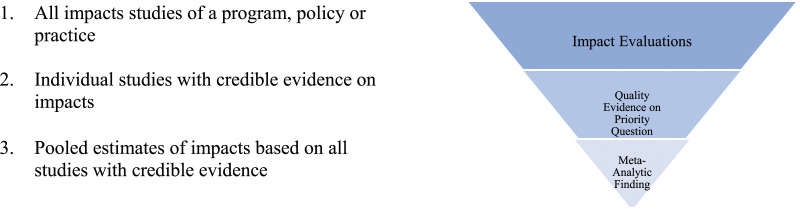
Evidence pipeline.

The core steps in the Meta-Analysis are straightforward. It begins with searching for all impact evaluations on the policy, practice or intervention in question, reviewing them to determine whether each study was designed and implemented in a way that produces credible answers to the questions of interest, and then pooling credible findings for the outcomes of interest (Figure 1). The details are more or less complex and the findings more or less useful depending on the subject of the analysis and the volume and quality of the available evidence.

For decades Meta-Analysis has been applied widely in medicine to combine evidence from multiple similarly designed studies of treatments evaluated on different samples of patients. Meta-Analysis works reasonably well in this setting, as much of the underlying research entails small randomized controlled trials of patient responses to various medical procedures or drug therapies for treating specific conditions. In these contexts, impact studies generally use common outcome measures, treatment variation is tightly controlled, and the most likely confounders (e.g., co-morbidities, severity of condition) can be controlled statistically. In contrast, Meta-Analyses in the social sciences commonly are more complicated due to their less clear objectives, greater variability in contexts, and lesser ability to control implementation fidelity. These differences can increase the tedium and complexity of conducting Meta-Analyses in the social sciences and render the findings more tenuous.

Use of Meta-Analysis in the social sciences accelerated around the turn of the century in response to evidence-based policy initiatives in the U.S., Canada, and parts of Europe (Campbell Collaboration, n.d; Davies et al., 2000; Donaldson et al., 2015; European Commission, n.d; Haskins, 2018; Margolis & Haskins, 2015; Nutley et al., 2007; Owen et al., 2022; Stack, 2018). In turn, the evidence-based policy initiatives sparked public and private efforts to accelerate the production and curation of evidence on the effectiveness (or lack thereof) of both current and alternative approaches for improving outcomes in education, workforce development, social welfare, and criminal justice.

Federal agencies and philanthropies made strategic investments in evidence building using randomized controlled trials and carefully designed quasi-experimental evaluations and agencies were created to support production and use of credible evidence. For example, the U.S. has an Office of Evaluation Sciences that works with Chief Evaluation Officers in the Departments of Education, Labor, Health and Human Services and Juvenile Justice to commission and use rigorous evidence to guide public policy decisions. These Federal Departments, as well as some university and nonprofit centers, have codified standards for credible evidence of effectiveness aimed at particular end-users. And there has been a proliferation of evidence review initiatives and platforms created for sharing curated evidence, typically including judgments about its credibility (Table 1).

**Table 1.** Selected Evidence Review Platforms.

Clearinghouse Name	Sponsoring Agency	Weblink
Best Evidence Encyclopedia	Johns Hopkins University’s Center for Research and Reform in Education (CRRE)	https://bestevidence.org/
Campbell Collaboration	Campbell Collaboration	https://www.campbellcollaboration.org/better-evidence.html
Center for Evidence-based Crime Policy	George Mason University	https://cebcp.org/evidence-based-policing/what-works-in-policing/research-evidence-review/
Clearinghouse for Labor Evaluation & Research (CLEAR)	U.S. Department of Labor	https://clear.dol.gov/
Collaborative for Academic, Social, and Emotional Learning	Collaborative for Academic, Social, and Emotional Learning (CASEL)	https://pg.casel.org
Crime Solutions	Office of Justice Programs	https://www.crimesolutions.gov/
Education Endowment Fund Evidence Reviews	Education Endowment Foundation (EFF)	https://educationendowmentfoundation.org.uk/education-evidence/evidence-reviews
Evidence for ESSA	Center for Research & Reform in Education, Johns Hopkins University	https://www.evidenceforessa.org/
Home Visiting Evidence of Effectiveness (HomVEE)	U.S. Department of Health & Human Services, OPRE	https://homvee.acf.hhs.gov/outcomes
National Clearinghouse on Families & Youth	National Commission on Families & Youth	https://ncfy.acf.hhs.gov/
Office of Juvenile Justice and Delinquency Prevention Model Program Guide	Office of Juvenile Justice & Delinquency Prevention	https://ojjdp.ojp.gov/evidence-based-programs
Pathways to Work Evidence Clearinghouse	U.S. Department of Health & Human Services, ACF	https://www.pathwaystowork.acf.hhs.gov/find-interventions
Teen Pregnancy Prevention Evidence Review Database	U.S. Department of Health & Human Services	https://youth.gov/evidence-innovation/tpper/studies-search
Title IV-E Prevention Services clearinghouse	U.S. Department of Health & Human Services	https://preventionservices.acf.hhs.gov/
What Works Clearinghouse (WWC)	U.S. Department of Education, Institute of Education Sciences	https://ies.ed.gov/ncee/wwc/
Workforce Systems Strategies	U.S. Department of Labor, Employment & Training Administration	https:/strategies.workforcegps.org/

Source: Program websites links were accessed November 13, 2023. Many of these review platforms also include guidelines on their review procedures and standards for included studies.

As the volume of credible evidence has expanded, it is increasingly common for evidence review platforms to include not only individual study reviews but also Meta-Analyses of findings on particular programs, policies or practices. These platforms have simultaneously made it easier to locate credible evidence relevant for Meta-Analysis while also exposing challenges using Meta-Analysis to guide policy and practice. For example, more widespread use of Meta-Analysis has revealed the degree to which the effectiveness of social policies and practices vary depending on the “three Cs”—client characteristics, service contrast and context (Weiss et al., 2014) Heterogeneity of impacts reduces the value of Meta-Analytic average impact estimates for the synthetic population represented by the study samples, while opening up the possibility of using the observed heterogeneity of impacts to judge the generalizability of findings to populations and implementation conditions of interest. This variation also can provide insights as to the mechanisms that affect whether impacts occur and, if so, their magnitudes.

Increased use of Meta-Analysis in the social sciences has highlighted the frequency with which analysts face challenges selecting focal outcomes and harmonizing the particular measures reported in individual studies. Often there are multiple and sometimes competing outcomes of interest and even the same outcome may be measured in different ways across studies. Sometimes it is important to measure potential unintended outcomes in addition to measuring the intended outcomes. For example, teen pregnancy prevention programs could reduce the incidence of teenage pregnancies and births while increasing sexually transmitted diseases and abortions. Workforce development programs could simultaneously improve employment rates and earnings of parents while reducing access to public support for health care and quality childcare. Furthermore, outcome metrics that carry a common label may have different interpretations depending on the reference population. For example, consider the interpretation of a 5-percentage point reduction in high school dropout rate for an intervention focused on an entire cohort of high school students versus for an intervention focused on chronically absent students. Intervention developers may prioritize learning whether the intervention works as planned, while a policy maker would prioritize knowing the average cost-effectiveness of the intervention (e.g., the cost per percentage point reduction in the dropout rate).

These factors need to be addressed when delineating the research questions and defining focal outcome measures and acceptable metrics for use in a Meta-Analysis. Regrettably, there rarely is a sufficient number of studies and detail on heterogeneity among the samples, interventions, and outcome measures to fully comprehend the implications of the variation for the applicability and generalizability of the findings. A common default practice is converting various measures of a common construct to effect size measures, which can be problematic when there is heterogeneity among the reference populations and samples that have been studied (Dong et al., 2008). For example, a .2 standard deviation change in earnings for a sample of low-income adults will be a much smaller average dollar wage gain than a .2 standard deviation change in earnings for a representative sample of the entire adult population.

Finally, prospective end-users of economic and social program and policy evaluations and evidence syntheses have diverse interests. Intervention developers tend to prioritize evidence that supports their understanding of whether something works and nuances of the critical features that affect how well, under what conditions and for whom it works. In contrast, policymakers and practitioners are more interested in how well interventions work with particular populations and in particular settings and the essential conditions for achieving maximum performance.

It is easy for studies designed with one audience in mind to ignore the evidence needs of another. For example, studies that simply focus on whether state support for after school programs improves student outcomes are helpful to state policy makers. Yet, they are unlikely to be useful to school staff deciding how to design and improve their own after school programs unless accompanied by details on the conditions and features of programs that are beneficial to students like theirs.

## Optimizing Use of Meta-Analysis Using the Current Evidence Base

A well-designed Meta-Analysis can be useful in five ways: it (1) identifies the corpus of potentially relevant evidence; (2) sorts that evidence by its credibility and relevance for estimating impacts on one or more outcome of interest; (3) standardizes the metrics for estimates of impacts and measurement error; (4) reports the study-level and pooled impact estimates for all studies; and (5) reports an estimate of variation in impacts across studies. Sometimes Meta-Analyses also have sufficient data to explore moderators of impact and/or impacts for particular subgroups of participants or programs (e.g., Durlak et al., 2011; Lundahl et al., 2006; Tipton & Olsen, 2018; Weiss et al., 2022).

For example, Weiss et al. explore the variation in the impacts across 30 large scale evaluations of college access programs, some of which included multiple treatment arms. The weighted average impact was sizeable and positive, but estimates varied significantly depending on the number of program components and what specific practices were used. Durlak et al. examined variation in impacts across 213 “universal social emotional learning” programs and found significant variation associated with whether or not programs included particular practices or experienced serious implementation issues. Lundahl et al. explored variation in impacts across 63 studies of parent training interventions and found variation associated with the mode of training delivery, whether the children were concurrently in therapy, and the economic status of families. Even in reviews like these with large numbers of studies, moderator analyses are necessarily exploratory since they lack the ability control for potential confounding influences. Yet, they fill “gaps in the evidence maps” that can inform policy development, program improvement, and/or future evaluations.

There are four pillars of best practices in Meta-Analysis: (1) the relevance of the questions addressed; (2) the credibility of the source evidence; (3) the comparability and applicability of the measures and metrics; and (4) the accessibility and use of the findings. Weakness in any of the pillars adversely affects the overall usefulness of the product.^3^ Over the past 20 years, the evidence base and infrastructure to support Meta-Analysis has opened doors for more and better use of evidence. The following are suggestions for building on the current infrastructure to further expand and improve the use of evidence.

### Focus on Important, Answerable Questions Grounded in Theory

Meaningful reviews focus on questions that have practical or policy relevance to one or more audiences—*practitioners* looking to improve their outcomes, *policy makers* looking to achieve a particular goal, or *social scientists* working to advance knowledge of some particular area. Often systematic reviews in the social sciences are motivated by the emergence of a corpus of data on a particular topic (e.g., the publication of a suite studies of workforce training programs prompted a Meta-Analysis by Peck et al. (2021) or emergent interest in a topic, such as the shortfalls in learning gains resulting from COVID that prompted a Meta-Analysis by Betthäuser et al. (2022). However, many Meta-Analyses are initiated by evaluators’ interest in achieving summative information on a particular issue such as the benefits of early education interventions (Camilli et al., 2010) or identifying factors that are predictive of the magnitude of benefits from cognitive-behavior programs for individuals in the criminal justice system (Landenberger & Lipsey, 2005; Lipsey, 2009).

It is helpful in prioritizing questions for study to consider who other than the primary audience for the study may also have interest in the findings. Although addressing the interests of this broader audience may require modifying analysis plans, making those modifications may substantially expand the usefulness and use of study findings. Broadening the study focus could reveal unintended benefits or adverse outcomes and increase understanding of the conditions under which the study findings are likely to apply to particular contexts of interest. For example, the prospective audience for findings on social emotional learning programs targeted at middle schoolers include professionals who develop social-emotional learning programs, middle school teachers, district education leaders, and federal policy makers. Groups will have varied levels of interest in the average effect of the many school-based programs that have been evaluated versus the average impact for different types of programs, programs targeted on particular grade levels, programs similar to those used in their districts, or the lower versus higher cost programs. Recognizing that research funders’ interests may trump interests of other groups, it is almost certain that devoting some effort to a broader audience will lead to more useful and better used studies.

### Design Reviews That Align With Existing Standards of Evidence in Various Policy Areas

This requires producing a clear description of the program, policy or practice(s) being evaluated and the acceptable comparison conditions, identifying the populations and outcomes of interest to the various constituent groups, and using metrics and timeframes for outcome measures that are meaningful to target audiences for the study findings. In addition to being good practice, this will minimize spurious variation in impact estimates across reviews that impede the ability of various constituent groups to make informed decisions regarding the relevance of findings for them. Over time, this investment in better study designs will strengthen the evidence eco-system by encouraging better design and documentation of primary studies and by streamlining the study search, screening, and coding processes for Meta-Analyses, especially for pre-registered studies and those included in prior reviews.^4^

### Use Data and Analytic Methods That Produce Credible Evidence for Specific Questions

The data and methods for a Meta-Analysis will vary depending on the goals of the study. For example, studies like Weiss et al. (2022) are designed to estimate the average impacts of interventions that share a common goal, but that intentionally vary in measurable ways (e.g., the populations they serve, nuances in program design and implementation, and focal outcomes). By harmonizing measures of the intervention qualities, population characteristics of interest, and settings across studies, it was possible to explore the heterogeneity of impacts along a variety of dimensions in ways that inform both theory and judgments about applicability and generalizability of the findings.

Other studies, like Betthäuser et al. (2022) and Littell et al. (2021) focus on the consequences of naturally occurring and planned interventions, respectively, that may vary in their impacts because of differences in the target populations and the nature and context of the interventions themselves. For example, Betthäuser et al. estimated the impacts of a naturally occurring intervention, COVID-19, on student engagement and learning. The primary goal was to learn from the variation in impacts across population groups and settings rather than to produce an average causal impact estimate. There was no option for an experimental evaluation to measure the impacts of the pandemic. However, since under normal conditions learning trajectories are quite stable, it is reasonable to assume that comparing trends before and during COVID-19 would yield plausible impact estimates.^5^ Because the studies in this Meta-Analysis used comparison group designs, it was especially important that the Meta-Analysis include a robust “risk of bias” assessment. This entailed looking for evidence of weaknesses in the sample or analysis that could adversely affect the credibility of the study findings, such as sample selection, model misspecification, and shifts in measurement strategy Sterne et al. (2016).^6^

Littell’s primary focus was on the generalizability of pooled findings from studies of multisystemic therapy (MST) conducted in many different countries, with a wide range of population groups and considerable natural variation in the intervention protocols. MST is intentionally adaptive to individual circumstances and the targeted outcomes vary across individuals depending on their presenting conditions. Thus, both MST services and their domains of impact vary depending on the needs and current status of recipients, complicating how one should design and interpret findings from a Meta-Analysis (Littell et al., 2021 & Chapter 4). Thus, for interventions like MST it is especially important to examine heterogeneity of impacts across outcome measures, as well as across qualities such as the reference population, implementation features and setting.

“Multisystemic therapy (MST) is an evidence-based treatment originally developed for youth with serious antisocial behavior [and] at high risk for out-of-home placement and their families…[and] subsequently adapted to address other challenging clinical problems experienced by youths and their families” (Henggeler & Schaeffer, 2016).

The main goal of Weiss et al. (2022) was to identify explanations for the variation in impacts of community college support services programs across programs. On average, participants in the 33 study samples for which impacts on college credits could be estimated earned significantly more credits than their “usual services” counterparts. However, estimates range from an average gain of more than 4 credits to an average loss of .5 credits. The Meta-Analysis intentionally focused on the relationship between impacts on credit accumulation and program design and implementation features.

These questions are aligned with the underlying theory of change for these programs. However, the analysis is exploratory since the program design and implementation features were not randomized. In contrast, estimated impacts for subgroups of the study samples identified by baseline characteristics have strong causal warrant. In this case, the ability to pool micro-data afforded a stronger analysis of impact variation associated with background characteristics such as gender and age than is possible when only study level data are available.

### Attend to the Comparability and Applicability of Outcome Measures

Meta-Analyses intended to guide policy and/or practice should use comparable outcomes measures or have a strategy for helping the end-user make meaning of the differences. For example, a study focused on improving overall academic achievement should use only evidence from studies that have used a global measure of academic achievement. Studies that measure only math achievement typically would not be appropriate for inclusion in a review focused on overall academic achievement but would be appropriate for a companion review of evidence on strategies for improving math achievement.

Similarly, estimates of impacts on focal outcomes measured at widely different intervals relative to treatment should not be treated as equivalent in the analysis. It is good practice to Meta-Analyze these separately or to include duration of follow-up as a moderator in the analysis and then report impact estimates by duration of follow-up. Finally, it is not wise to blindly assume that converting measures like standardized test scores based on different tests to Z-scores yields consistent, interpretable indicators of the magnitude of impact; they only provide reliable estimates of the statistical significance of the sample impact estimates.

### Disseminate Useful Evidence in Accessible Formats

Meta-Analysis is a means of sensibly identifying and combining the findings of multiple studies focusing on a common goal. However, too often the results are either ignored or used selectively when the findings fail to support prevailing beliefs and wishes. Focus, format and accessibility of evidence affect its use and usefulness. It is critically important to include all credible evidence in reviews and to provide access to individual study details—information that is necessary, but not always sufficient to judge the applicability and generalizability of findings and to identify gaps in what we know.

“Unlike statistical significance (p-values), effect sizes represent strength of relationships without regard to sample size.” (Hedges, 2008)

Meta-Analysis findings can be especially useful when there are many well-designed studies looking at the effectiveness of a policy or practice, such as improving social-emotional well-being of students (Durlak et al., 2011), preschool outcomes (Joo et al., 2020), or employment and earnings of participants in career pathways programs (Peck et al., 2021). These types of reviews focused on “hot button” issues and tend to be widely cited in academic literature and the press.

Despite strong investments in improving the quantity, quality, and dissemination of evidence reports and evidence reviews, practitioners are still not big consumers of Meta-Analyses (Tseng & Coburn, 2019). Even “deep” users of evidence tend to favor evidence that is designed to *“help them achieve their goals* …*[or] explain choices”* (Farley-Ripple et al., 2022) over detailed research reports or summaries of the numbers in those reports. Brief, accessible summaries like that on the Peck et al. Meta-Analysis of career pathways programs (Strawn et al., 2021) complement the evidence review information housed on the various evidence review platforms (see Table 1 above).

Useful and used evidence begins with focusing studies of useful questions for the target audience. This may require multiple layers of analysis and findings that vary in their interest to prospective end users groups. For example, the evidence needs of a policy maker focused on how to allocate scarce resources to maximize poverty reduction among parents of young children differ from those of a community-based organization trying to improve job placement rates and quality for program participants. The former will be more interested in evidence on the effectiveness and costs of distinct strategies for achieving particular outcomes—for example, through employer-based training initiatives or financial supports to encourage unemployed and low-wage workers to enroll in career and technical training. The latter is more likely to be interested in the benefits of programs designed and implemented under their control—for example, recruitment and onboarding strategies, variations in durations and intensities of training, supplemental support services. A systematic review that compiles average program impact estimates and Meta-Analyzes them without attention to the nuanced interests of target audiences will likely not serve any audience especially well.

It is very helpful to report supplemental descriptive information aligned with the interests of prospective constituents, even though it may not have been integral to the current evaluation. Such information includes features of the study sample, settings, intervention design and implementation, dosage, and secondary outcomes and their estimated impacts. This makes it easier for future analysts to assess what more can be learned from extant evidence, and it signals to others the types of descriptive information needed to smartly judge the likely relevance of study findings to policy makers and practitioners. This will improve the likelihood that future research will plug current evidence gaps.

## Room for Improvement

It is, of course, important to continue to increase the number and quality of impact evaluations. However, it is equally important to design, conduct, and report future evaluations in ways that allow future Meta-Analyses to address higher order questions about the cost of achieving impacts and the applicability and generalizability of findings. At a minimum, this requires including cost analyses in evaluations, making studies more discoverable and reporting more inclusive.

Over the last two decades, there has been increased attention to inclusion of cost analysis as a core component of program and policy evaluations. For example, the Institute of Education Sciences incorporated cost analysis into its Standards for Excellence in Education Research (https://ies.ed.gov/seer/index.asp), user-friendly guidelines have been produced by a panel of experts and widely disseminated (Cost Analysis Standards Project, 2021) and the U.S. Department of Education funded a Cost Analysis in Practice Project to provide on-line training modules and resources (https://capproject.org/modules-welcome).

Over the last two decades, guidelines and tools for conducting and disseminating Meta-Analysis have improved, and the workforce of skilled Meta-Analysts has expanded. Concurrently, the pace of production and quality of Meta-Analyses have increased. Yet, there is still considerable room to improve the availability, use and usefulness of Meta-Analysis through tweaks to individual study design and reporting and strategic expansion of both the evidence base and capacity to efficiently conduct and update systematic reviews and Meta-Analyses.

We have good guidance on the mechanics of using Meta-Analyses to produce unbiased estimates with minimum estimation error, explore heterogeneity in impacts and outcomes across studies, population groups and implementation contexts, and report average impacts and impacts for subsets of studies with particular properties. In contrast, we have relatively few examples of deliberate efforts to assess the generalizability of study findings. The major evidence review platforms (see Table 1 above) emphasize reporting individual study findings and computing average impact estimates across studies that meet established inclusion criteria. A few platforms (e.g., the WWC) have begun integrating Meta-Analyses into their platforms. However, evidence review platforms largely ignore differences that may affect replicability, reproducibility, and cost-effectiveness. For example, they tend not to have standards for reporting on reference populations, settings, implementation, and duration of follow-up (National Science Foundation & Institute of Education Sciences, 2018). One reason is that there are very few clusters of well-designed, implemented, and reported studies to support exploration of generalizability of the findings. Below are recommendations for mitigating this shortfall through improving the design and reporting of individual studies and by routinely taking advantage of state of art methods and tools for designing, conducting and disseminating Meta-Analyses in ways that would improve generalizability and applicability of individual studies or sets of studies.

### Improve the Design and Reporting of Individual Studies

There are at least three ways to improve the applicability and generalizability of impact findings other than doing more studies. One is to **align the study design with the implementation context and a well-grounded theory of change**. A second is to **include serious implementation research** in the study, and the third is to **improve the accessibility of study findings and details**. To this end, it can be helpful for those doing impact evaluations in the social sciences to follow guidelines such as Common Guidelines for Education Research and Development (Earle et al., 2013) and Companion Guidelines on Replication and Reproducibility (National Science Foundation & Institute of Education Sciences, 2018). These guidelines prompt designers of the primary research studies to think more broadly about the prospective applications of study findings and how minor tweaks to study design, analysis and reporting protocols may improve the usefulness and use evidence.

Well-designed studies build on extant knowledge of target populations, settings, and interests of prospective consumers. As with the axiom “*measure twice and cut once*,” it is best to **work from grounded theory to research questions and then to the study design**. This then lays the foundation for data collection, analysis and reporting that is more likely to be useful and used by target audiences for the study and in subsequent Meta-Analyses.

These principles are core for improvement science and rapid cycle evaluations (Bryk et al., 2015; Hargreaves, 2014; Maynard et al., 2022) and realist reviews (Pawson, 2002). However, they have not been routinely adopted in Meta-Analysis of impact findings even though doing so would be an easy way to improve the design of the analysis and the interpretation and communication of findings. Favorable findings aligned with a pre-specified theory of change offer intuitive rationales for the observed outcomes. When findings are null or adverse, the theories of change also support exploration of the reasons and potential modifications to the theories and/or the programs, policies of practices themselves.

Anchoring impact evaluations in formal theories of change improves the prospects that evaluation designs focus on priority outcomes for both practitioner and policy audiences. In turn, this improves the prospects that studies will collect and report information critical for designing and conducting impactful Meta-Analyses. This includes making wise decisions about the outcomes measured and metrics used as well as complementary qualitative and quantitative information to guide meaningful application of the findings in policy and practice settings.

“Navigating this dizzying array of constructs can be a challenge even for the seasoned researcher.” (Lazowski & Hulleman, 2016, p. 3)

Including **targeted implementation research** in impact evaluations can be critical for judging the applicability and generalizability of findings and the appropriateness of pooling evidence across studies. For example, product designers and developers likely will be most interested in evidence to support product marketing or production efficiency while policy makers and practitioners will be more interested in evidence about effectiveness in improving specific outcomes for particular population groups (e.g., improving earnings of low-wage workers or graduation rates among academically struggling youth). However, both groups will need information on program design, implementation, and implementation context to make meaning of Meta-Analytic findings and to gauge whether the findings are likely to generalize to their context.

Thoughtful measurement and use of data on the characteristics of study samples, settings, and intervention features can improve the precision of impact estimates and judgments about their generalizability of individual study findings. Then, including the contextual information in Meta-Analyses improves the precision of impact estimates and the ability to make better informed decisions based on the available evidence. For example, Olsen et al. (2023) illustrates methods for using this information to make informed “guesstimates” tailored to priors and preferences.

Finally, in order to reap the benefits of better designed studies with complementary implementation information, evaluators need to **make detailed study information accessible for Meta-Analysis**. One of the best ways is to normalize pre-registration of studies on platforms that share access to study details and results outside of paywalls. Fortunately, there is now a robust and constantly improving infrastructure to support this. Pre-registration platforms like the OSF Registries),the AEA RCT Registry and the Registry of Education Effectiveness Studies (REES) also can and do serve as repositories for reports, technical documentation, and data (in controlled- or open-access).^7^

Pre-registration of studies serves multiple goals. First, it makes it possible for interested researchers, policy makers, and practitioners to learn of studies, whether in process or completed. Second, pre-registration prompts evaluation teams to carefully consider critical aspects of study design up front, including prospective audiences for the findings and their information needs. Third, it provides a means of monitoring adherence to protocol and the integrity of deliberate modifications plans. Fourth, when evaluation designs are modified mid-stream, pre-registration provides an accessible means of learning and being able to judge the legitimacy of modifications in study designs. Fifth, it prompts evaluators to plan for a responsible data sharing plan.^8^

At a minimum, all primary studies should report data on the populations represented in the study sample, the qualities of the interventions evaluated, the nature of the counterfactual conditions, the outcomes examined and the timing of those measurements, and the timing of the study and its setting.^9^ This allows advance judgments about whether questions can be addressed, the advantage (or lack thereof) of conducting a Meta-Analysis to explore pattern of impacts an judge what observed variation might suggests about the applicability and generalizability of the study findings across population groups and/or implementation contexts (Forero et al., 2019; Methods Group of the Campbell Collaboration, 2017; Page et al., 2021; Gordon et al., 2023).

### Use State-of-the-Art Meta-Analysis Guidelines and Tools and Make Key Intermediate Products Publicly Accessible

Good Meta-Analyses begin with a systematic search for available evidence on a particular topic. The results define the corpus of evidence appropriate for inclusion in the planned Meta-Analysis, establish bounds on the questions that can be addressed and identify seemingly related evidence that should be excluded from the review and the reasons why (Owen et al., 2022; Slavin, 2020).

It is important to **take stock of the available evidence and consider the implications for study focus and design**. In cases where there are only a few relevant studies, the evidence may be most useful for sharing in a systematic review that simply describes the studies and presents the individual study findings. If the review includes average impacts across the few studies, it would be appropriate to estimate “fixed effects” (Borenstein, 2019) and the authors should alert the reader to the nature of the few samples, settings, and implementation contexts represented in the analysis.

Williams et al. (2022) is a rare example of a Meta-Analysis designed from an implicit logic model built on extant theory. The team designed evidence screening and abstraction process that captured data on not only key outcomes and treatment status but also characteristics of the sample, key qualities of the treatments and the settings, and methodological strategies that could affect impact estimates. The resulting analysis sample included large 191 studies, 1109 effect sizes, and data for more than 250 thousand students. Thus, the study was able to move beyond an estimating the average impact for the pooled sample and systematically explore heterogeneity of impact estimates in ways that informed judgments about generalizability and applicability of findings to particular populations, intervention features and settings (Williams et al., 2022, Table 1, page 593).^10^

In cases where the number of studies hits mid-range (e.g., 15 to 20 or more), it may be reasonable to explore the degree and potential sources of variation in impacts across studies—a first step toward examining the likely generalizability of findings beyond the study samples. This should entail using a “random effects” model to estimate the mean impact and test whether there is significant variation in impacts across studies (Borenstein, 2019, Chapter 9; Pigott & Polanin, 2020; Jaciw et al., 2022). Then, in cases where there are large numbers of studies of a particular issue and the supporting contextual data on populations, settings, and implementation, one also might choose to use Meta-Regression models to assess the extent to which variation in impacts is explained by factors such as the nature of the program, policy, or practice, the setting in which it was tested and/or the characteristics of the reference populations (Borenstein, 2019, Chapter 13).

Depending on the types and consistency of contextual data available for studies, it can be productive to **conduct separate analyses for subgroups of studies or Meta-Regression analysis** to identify factors that are associated with stronger or weaker impacts of the focal program, policy of practice under study. Even though Meta-Regressions of this nature generally do not produce causal evidence, they can be very helpful in interpreting findings and refining theories.

**Figure 2.**
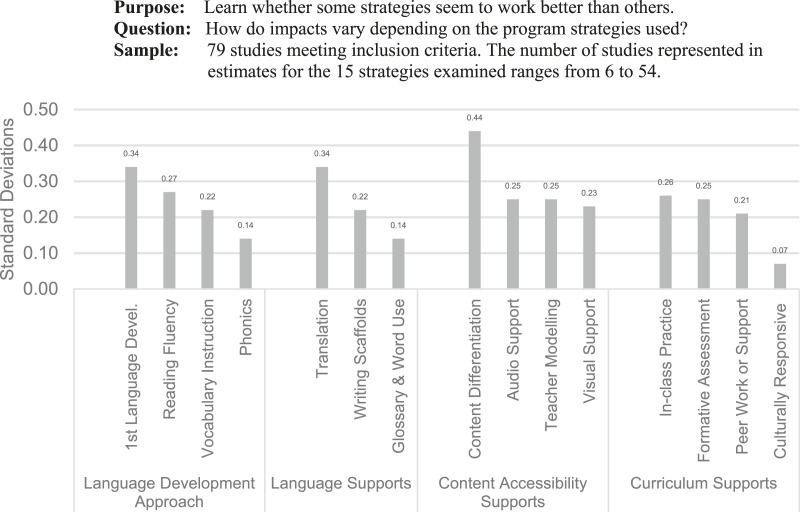
Impacts on Language Development for English Language Learners by Program Strategies. Source: Adapted from Williams & Garrett, (2023), Slides 18 & 19 with permission of Authors. Notes: Estimates are regression adjusted for study and methods characteristics. All impact estimates except those for phonics and culturally responsive supports are statistically significant.

As an example, a recent Meta-Analysis of programs for English Language Learners explored the relationship between program effectiveness and four specific program features: approaches to language development, language supports, content accessibility, and curriculum supports (Williams & Garrett et al., 2023). A quick look at the variation in impact across studies using different approaches in each of the four domains of practice reveals considerable variability in the magnitudes of effects across programs using different strategies and supports (Figure 2). These patterns can be further explored in the context of the program logic model and variation in characteristics of the settings and students represented in the programs with various program features.

In the rare case when there is micro data for multiple impact studies focused on a common outcome and using reasonably well aligned measures of outcomes and contextual factors, it may be productive to analyze the pooled data. Doing so has the potential to capitalize on both within- and cross-study variation in characteristics of the study samples and interventions to learn about predictors of variation in impacts.

**Figure 3.**
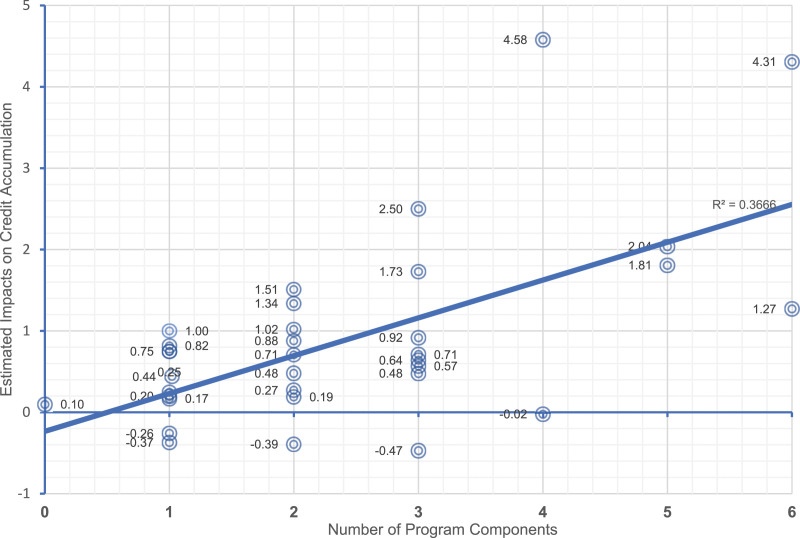
Exploring Variation in Impacts: Micro-Regression Results from THE-RCT. Source: Adapted from Weiss et al. (2022) with permission. Note: Impact estimates can be interpreted as causal. However, differences in impacts associated among programs with different numbers of program components are correlational, since programs were not randomized to different numbers or types of components.

The Higher Education Randomized Controlled Trials data repository (THE-RCT) illustrates the benefits of shared access to microdata for such purposes (Diamond et al., 2023). THE-RCT provides access to micro-datasets for 30 randomized controlled trials evaluating 39 different strategies for improving persistence and credit accumulation of community college students. All of the evaluations used administrative data for the key outcome measures and included coded data on program components and their intensities. This makes it possible to examine heterogeneity of impacts across programs that vary in the types and qualities of services offered.^11^ For example, Weiss et al. (2022) used the prevalence and number of components to create a measure of “comprehensiveness” (Figure 3, horizontal axis). The study team interacted the number of components with Treatment Status to estimate impacts for participants who were exposed to different numbers of components (Figure 3, vertical axis).

The results show a very strong relationship between the number of components and program impacts. However, because program participants were not randomly assigned to programs with different numbers of components, they demonstrate only a strong correlation, not a causal relationship. The same dataset could be used to examine the extent to which other factors such as participant characteristics or settings explain variation in impacts. As the number of rigorous impact evaluations grows and data sharing opportunities of this type expand, these types of studies will be more common and more informative.

## Conclusions

Meta-Analysis is a very useful method for maximizing the usefulness of program and policy evaluation. It does the hard work of screening for relevant studies with credible findings to inform policy, summarizing the findings in a logical, orderly manner and, where possible, expanding knowledge about the size, variability, and reliability of estimated impacts and how estimated impacts vary within and across contexts. In that way, they facilitate the process of assessing applicability and generalizability.

The growing number of studies makes search and screening tasks more difficult. However, other factors make the job easier and the findings more useful. We have an increasingly robust evaluation workforce that is familiar with search resources and strategies and with evidence guidelines that are important for Meta-Analysis. There have been major improvements in the quality of impact evaluation designs and reporting of key features of the design, sample, implementation, and analytic methods in study reports. More studies are being pre-registered on accessible platforms, and more reports and datasets are being shared through restricted or open access platforms. We are making more and better use of technology for tasks ranging from searching for studies through analysis and data sharing. This work will continue to improve and be more impactful as evidence bases continue to grow and improve and new supports continue to roll out and their use expand.^12^

## Notes

1. See U.S. Department of Education (n.d.) “Post Secondary Student Success Program.” Accessed at: https://www2.ed.gov/programs/pssp/index.html.

2. Only $99 million of the I-3 funding was spent on the mandated studies, which were conducted by independent evaluators.

3. Having credible answers to important questions does not ensure they will guide the actions of policymakers or practitioners. Despite concerted efforts in the U.S. for more evidence-based policy making in education, as of 2019, much of the effectiveness research on education policies and practices that was publicly funded over the prior 15 years was not being used by policy makers for various reasons. These include shortfalls in the breadth of the evidence; failure to produce “trusted, impartial, educator-friendly reviews of the research”; and/or lack of incentives for educators and providers to alter course based on the evidence (Slavin, 2019).

4. Most evidence review platforms publish their review standards and some, like the What Works Clearinghouse (2022) and the U.S. Department of Labor (2022), share coded data. Moreover, increasing numbers of federal agencies (e.g., the Institute of Education Sciences) and philanthropic organizations (e.g., the Arnold Foundation) are requiring evaluators to pre-register their studies on platforms that encourage or require this type of detail.

5. Ideally, impact estimates would be generated using analytic methods such as interrupted time series or regression discontinuity modeling (Imbens & Lemieux, 2008; McDowall et al., 2019). However, Betthäuser et al. (2022) do not provide details of the underlying analytic models used in their included studies.

6. Weiss et al. (2022) do not discuss risk of bias in their Meta-Analysis, likely because all of the studies were large-scale randomized controlled trials that had been through numerous layers of peer review prior to selection for inclusion in their Meta-Analysis.

7. See Spybrook et al. (2022) and Appendix A for a more information on data archive options.

8. Neild et al. (2022) offers an example of available data sharing guidance.

9. These are commonly referred to as PICOTS—Patient/Population, Intervention, Comparison Condition, Outcomes, Timing, and Setting (Schultz et al., 2010).

10. This analysis was conducted using the Metafor package for R available at: https://metafor-project.org/doku.php/metafor.

11. All of the studies in Weiss et al. (2022) were conducted by MDRC. This Meta-Analysis included about 40% of the RCTs of similar programs identified by the What Works Clearinghouse through 2022. As one of the first social science research firms, MDRC has had a major influence on evaluation standards and practices such as those promoted in the NSF/IES Common Guidelines and on the expectations of public and private funders of program evaluation research (American Evaluation Association Evaluation Policy Taskforce, 2022; Margolis & Haskins, 2015).

12. There are exploratory efforts underway to apply Bayesian methods to Meta-Analyses (Walsh et al., 2023) and major developments to strengthen and accelerate Meta-Analysis. MetaReviewer, produced under a grant from the National Science Foundation, is an example of a recent software product that can improve the pace, quality, and use of systematic reviews conducted using Meta-analytic methods. This is a platform to design and manage Meta-Analyses from design through data collection, coding, analysis reporting and archiving (American Institutes for Research, n.d).

## Appendix A

**Table A1.** Options for data archiving.

**Dataverse.** https://dataverse.org/ Dataverse is an open-source platform for publishing data developed at Harvard University’s Institute for Quantitative Social Science. There are more than 340,000 data sets stored in Dataverses worldwide.
**ICPSR**. https://www.icpsr.umich.edu/web/pages/ ICPSR (Inter-University Consortium for Political and Social Research) archives research in the social and behavioral sciences and is a frequent option for OPRE projects. The archive offers Open ICPSR, an unrestricted access archive that does not require the same level of curation and cleaning as ICPSR.
**Federal Statistical Research Data Centers (FSRDCs).** https://www.census.gov/about/adrm/fsrdc.html
These are partnerships between federal statistical agencies and research institutions. FSRDCs provide secure environments to support researchers using restricted-access data.
**National Archive of Criminal Justice Data** https://www.icpsr.umich.edu/web/pages/NACJD/ this archive houses data on crime and justice. The Bureau of Justice Statistics, the National Institute of Justice, and the Office of Juvenile Justice and Delinquency Prevention of the Department of Justice sponsor the NACJD
**National Data Archive on Child Abuse & Neglect** https://www.ndacan.acf.hhs.gov/ the ACF Children’s Bureau funds this archive, which includes data sets relevant to the study of child abuse and neglect. Data sets include data files, complete documentation, and technical support from the NDACAN staff.
**National Institute of Mental Health Data Archive** https://nda.nih.gov/ this archive facilitates data sharing across mental health and other research communities. It provides curated human subjects research and longitudinal storage of a research participant’s information generated by one or more research studies.
**Substance Abuse & Mental Health Services Administration** (SAMHSA) Data Archive https://www.datafiles.samhsa.gov/ the Center for Behavioral Health Statistics and Quality (CBHSQ) maintains this archive. CBHSQ has primary responsibility for collecting, analyzing, and disseminating SAMHSA’s mental health data.

Source: Gordon, H., O’Callahan, C., Hunter, K., Inanc, H., Grider, M., & Zaveri, H. (2023). Guide to Publishing Research Data for Secondary Analysis. Washington, DC: Administration for Children and Families, U.S. Department of Health and Human Services. OPRE Report #2023-227. https://www.acf.hhs.gov/opre/report/guide-publishing-research-data-secondary-analysis.
